# The biology of Hepatocellular carcinoma: implications for genomic and immune therapies

**DOI:** 10.1186/s12943-017-0712-x

**Published:** 2017-08-30

**Authors:** Galina Khemlina, Sadakatsu Ikeda, Razelle Kurzrock

**Affiliations:** 1 Department of Geriatrics, University of California, UC San Diego, 9500 Gilman Drive, #9111, La Jolla, CA 92093-9111 USA; 2 Department of Medicine, Division of Hematology/Oncology, and Center for Personalized Cancer Therapy, University of California, Moores Cancer Center, San Diego, USA; 3 Tokyo Medical and Dental University, Tokyo, Japan; 4 Kaiser Permanente Southern California, San Diego, USA

**Keywords:** Hepatocellular carcinoma, Next-generation sequencing, Molecular targeted therapyg

## Abstract

Hepatocellular carcinoma (HCC), the most common type of primary liver cancer, is a leading cause of cancer-related death worldwide. It is highly refractory to most systemic therapies. Recently, significant progress has been made in uncovering genomic alterations in HCC, including potentially targetable aberrations. The most common molecular anomalies in this malignancy are mutations in the *TERT* promoter, *TP53*, *CTNNB1*, *AXIN1*, *ARID1A, CDKN2A* and *CCND1* genes*.* PTEN loss at the protein level is also frequent. Genomic portfolios stratify by risk factors as follows: (i) *CTNNB1* with alcoholic cirrhosis; and (ii) *TP53* with hepatitis B virus-induced cirrhosis. Activating mutations in *CTNNB1* and inactivating mutations in *AXIN1* both activate WNT signaling. Alterations in this pathway, as well as in *TP53* and the cell cycle machinery, and in the PI3K/Akt/mTor axis (the latter activated in the presence of PTEN loss), as well as aberrant angiogenesis and epigenetic anomalies, appear to be major events in HCC. Many of these abnormalities may be pharmacologically tractable. Immunotherapy with checkpoint inhibitors is also emerging as an important treatment option. Indeed, 82% of patients express PD-L1 (immunohistochemistry) and response rates to anti-PD-1 treatment are about 19%, and include about 5% complete remissions as well as durable benefit in some patients. Biomarker-matched trials are still limited in this disease, and many of the genomic alterations in HCC remain challenging to target. Future studies may require combination regimens that include both immunotherapies and molecularly matched targeted treatments.

## Background

Malignant tumors of the liver are classified as primary or secondary (metastatic) [1]. Common primary malignancies of the liver are hepatocellular carcinomas (HCC), which represent approximately 90% of cases [2]. Cholangiocarcinoma is a bile duct neoplasm accounting for 10%–15% of primary hepatic malignancies [3]. The nosology of bile duct cancer includes the following: (i) intrahepatic cholangiocarcinoma (ICC), arising from the small bile ducts within the liver; (ii) extra-hepatic cholangiocarcinoma (ECC), originating from the extra-hepatic bile duct epithelium; and (iii) gallbladder cancer [4]. Fibrolamellar carcinoma is a rare primary liver tumor that represents only 0.85% of primary liver cancers, but is the diagnosis in 13.4% of young patients (under the age of 40) [5].

HCC is a major global health problem. From a worldwide perspective, it is the third most common cause of cancer-related mortality [6]. HCC has several known etiologic factors: hepatitis B, hepatitis C, alcohol use, non-alcoholic steatohepatitis, and obesity [7, 8]. The exception is fibrolamellar carcinoma, which is generally found in adolescents and young adults who have no underlying liver disease [9]. The risk factors for fibrolamellar carcinoma remain unknown [10]. Fibrolamellar carcinoma usually develops in a normal-appearing, non-cirrhotic liver. Patients diagnosed with fibrolamellar carcinoma have a better outcome than those with other forms of HCC [10]. Recent molecular research have shown a high prevalence of a fusion gene--DNAJB1-PRKACA (found in 79%–100% of patients diagnosed with fibrolamellar carcinoma) [10]. In addition, fibrolamellar carcinoma demonstrates strong expression of EGFR by immunohistochemistry [9, 11]. Various genomic features characterize the stages of the diverse types of HCC. (Table 1).

**Table 1:** Pathologic features and molecular signatures of liver lesions

Tumor type	Pathologic features	Molecular signatures
Focal nodular hyperplasia (FNH)	Well-differentiated hepatocytes [88]	IHC staining positive for CK19, NCAM in proliferating ductules [88]
	Intervening fibrous bands radiating from a central scar, Abundant, proliferating bile ductules	GS staining pattern: map-like pattern [89]
		No mutation of *APC, AXIN, CTNNB1, HNF1, TP53* [90–92]
Hepatocellular Adenoma (HCA)	Well differentiated proliferating hepatocytes in cords one to two cells thick and lacking portal tracts [88]	IHC staining positive for CK7 [88]
	Rare bile ductules	GS staining pattern: diffuse pattern (beta-catenin activating pattern) or absent/irregular pattern (beta-catenin normal pattern) [88]
	Naked arterioles	Loss/mutation of TCF1 gene that encodes HNF1 (35–40% of HCA) [93, 94]
		Activating mutation of beta-catenin (10% of HCA) [91, 94]
		Overexpressed SAA, CRP, and gp130 in inflammatory subtype (50% of HCA) [95]
Dysplastic nodules (DN)	Vaguely (low-grade DN) or distinctly (high-grade DN) nodular with peripheral fibrous scar [96]	TERT promoter mutation in 6% of low-grade DN; 19% of high-grade DN [97]
	Mild increase in cell density with monotonous pattern, with no cytologic atypia (low-grade DN) or increased cellularity in irregular trabecular pattern with moderate atypia (high-grade DN)	
	No pseudoglands or markedly thickened trabeculae	
	Unpaired arteries sometimes present	
	No stromal invasion	
Early HCC	Increased cell density with an elevated nuclear/cytoplasm ratio and irregular thin-trabecular pattern [98]	Increased mRNA expression of GPC3 and survivin and down regulation of LYVE1 [99–103]
	Varying numbers of portal tracts inside the nodule	Positive IHC staining for GS, HSP70, and GPC3 [104–107]
	Pseudo-glandular pattern	
	Diffuse fatty change	*TERT* promoter mutations in 61% of early HCC [97]
	Varying numbers of unpaired arteries	
	Stromal invasion present	
Fibrolamellar HCC	Arising in non-cirrhotic liver [11]	Fusion gene--*DNAJB1-PRKACA* [10]
	Nests of well-differentiated oncocytic cells in a background of acellular but dense collagen bundles arranged in parallel lamellae	Overexpression of EGFR [11].
Advanced HCC	Unifocal, multifocal, or diffusely infiltrative soft tumor [98]	Inactivation of *TP53* [108, 109]
	Polygonal cells with distinct cell membranes, abundant granular eosinophilic cytoplasm, round nuclei with course chromatin, and higher nucleus/cytoplasm ratio,	
	Tumor capsule present	Activating mutations of *CTNNB1* [108]
	Invasion and minute intrahepatic metastasis	Other alterations listed in Table 2
	Unpaired arteries	
	Absent portal tracts	

*Abbreviations* **:** *APC* Adenomatous Polyposis Coli, *CSN5* COP9 Signalosome complex (CSN), *CRP* C reactive protein, *DN* Dysplastic nodules, *DNAJB1*-PRKACA(DnaJ (Hsp40) Homolog, Subfamily B, Member 1- Protein Kinase, CAMP-Dependent, Catalytic, Alpha), *CTNNB1* Catenin Beta 1, *EGFR* Epidermal Growth Factor Receptor, *EpCAM* The epithelial cell adhesion molecule, *GS* Glutamine synthetase, *HCA* Hepatocellular adenomas, *HNF1* Hepatocyte Nuclear Factor 1, *HPCs* Hepatic progenitor cells, *HSP70* Heat-shock protein70, *GPC3* Encodes glypican-3, *IHC* Immunohistochemistry, *LYVE1* Lymphatic Vessel Endothelial Hyaluronic Acid Receptor 1, *NCAM* Neural Cell Adhesion Molecule, *SAA* Serum amyloid A, *TCF1* Transcription Factor 1**,** *TERT* Telomerase reverse transcriptase

For patients suffering from advanced, conventional HCC, chemotherapy failed to demonstrate a survival advantage [12]. The multikinase inhibitor sorafenib is the only agent that was approved by the Food and Drug Administration (FDA) for HCC [13]. It demonstrated a statistically significant, but modest, survival benefit in two large phase III trials [12, 14, 15]. The most frequent toxicities related to sorafenib are fatigue, nausea, weight loss, hand-foot skin reaction and rash, and diarrhea [16]. However, the prognosis for patients remains dismal because the response rate with sorafenib is less than 5 % [15] and the median overall survival is extended by only about 2.5 months [15]. There is no established, effective second-line drug beyond sorafenib. Hence, understanding the underlying biology of HCC and the development of innovative treatments for this malignancy is of paramount clinical importance [17].

## Genomic aberrations in HCC

We reviewed the genomic landscape for primary HCC (other than cholangiocarcinoma and fibrolamellar carcinoma) and implications for therapy. Interestingly, recent genomic sequencing identified 161 putative genetic alterations in HCC [18] (Table 2).

**Table 2:** Commonly aberrant genes in hepatocellular carcinoma

Gene	Aberration Frequency (% of patients)	Pathway	Function	Examples of Potential Targeted Agent^a^
*TERT promoter*	About 60% [46, 47]	Telomere maintenance	Adds telomere repeats (TTAGGG) onto chromosome ends, compensating for the erosion of protective telomeric ends that is a normal part of cell division [110]	
*TP53*	Mutations: 3–40% Loss: 2–15% [20–22]	P53 pathway	Tumor suppressor [23]	Bevacizumab [33]
			*TP53* gene regulates the expression of VEGF-A. Anti-angiogenic agents were correlated with longer PFS in patients harboring *TP53*- mutant tumors [32]	Ramucirumab [111]
				Sorafenib [13]
				Wee-1 inhibitors [36]
*CTNNB1*	Mutations: 11–41% [20–22]	Wnt pathway	Regulates cell adhesion, growth, and differentiation [23].	BBI608 is a potent small molecule inhibitor [112]
				PRI-724[112]
				Sulindac [113]
*AXIN1*	Mutations 5–19% [20–22]	Wnt pathway	Regulates cell adhesion, growth, and differentiation.	Small molecule inhibitor XAV939 [31]
*ARID1A*	Mutations 4–17% [20–22, 37]	Chromatin remodeling	Transcriptional activation and repression of selected genes via chromatin remodeling [23]	
*CDKN2A*	Deletion 7–8% [18, 20]	Cell cycle	Tumor suppressor gene promotes cell cycle arrest in G1 and G2 phases. Suppresses MDM2 [23]	CDK4/6 inhibitor palbociclib [13]
*ARID2*	Mutations-5-7% [18, 21, 37]	Chromatin remodeling	Tumor suppressor gene with a role in the transcriptional activation and repression of selected genes [23]	
*RPS6KA3*	Mutations 4–7% [18, 22]	Dual function-regulation of the MAPK/ERK and mTOR signaling	Mediates stress-induced and mitogenic activation of transcription factors and cellular differentiation, proliferation, and survival [23].	
*CCND1*	Alterations (focal amplifications or deletions) 4.7%–7% [18, 41]	P53 pathway Cell cycle	Functions as a regulatory subunit of CDK4 or CDK6, whose activity is required for cell cycle progression [23].	Palbociclib [13]
*FGF3*, *FGF4* or *FGF19*	Alterations (focal amplification or deletions) 4–5.6% [18, 41]	FGF pathway	FGF family members possess broad mitogenic and cell survival activities and are operative in tumor growth and invasion, and tissue repair [23].	Brivanib [44]
				[114]
				BIBF 1120 [114]
				Dovitinib [114]
				Lenvatinib [114]

^a^For many of these targeted agents, it is not yet clear if use of the agent in patients with the cognate aberrant genes is effective

Assessment of genomic portfolios defined three groups of genes related to risk factors as follows: (i) *CTNNB1* with alcoholico; (ii) *TP53* with hepatitis B virus (HBV) induced cirrhosis; and (iii) others that do not have a distinct pattern, mainly in patients with hepatitis C virus (HCV) infection, metabolic syndrome, and hemochromatosis [18]. Analyses according to tumor stage demonstrated that *TERT* promoter mutation was seen more frequently in early-stage tumors. On the other hand, *TP53* and *CDKN2A* alterations, as well as amplification of the chromosome 11 amplicon that encodes *FGF3, FGF4, FGF19* and/or *CCND1* were observed more commonly in advanced stages [18].

## Signaling pathways

Recent data have identified several abnormal signaling pathways in liver carcinogenesis. Some of these aberrant signals provide a potential source of novel molecular targets for new therapies [19].

### Wnt pathway

#### CTNNB1

Approximately 11–41% of liver malignancies harbor *CTNNB1-*activating mutations [20–22]. The beta-catenin protein CTNNB1 is an important component of the canonical Wnt signaling pathway [23]. The encoded protein product anchors the actin cytoskeleton and may be responsible for communicating the contact inhibition signal that causes cells to halt division once the epithelial sheet is complete [23]. Delgado and coworkers demonstrated a complete response in a mouse model of hepatic carcinogenesis after suppression of β-catenin [24]. Several other inhibitors of this pathway, in particular WNT inhibitors, are also being tested in clinical studies, and include, but are not limited to, PRI-724 (NCT02195440) and BBI608 (NCT02279719). Further, accumulated evidence suggests that non-steroidal anti-inflammatory drugs (NSAIDs) such as celecoxib and sulindac also impact the Wnt/ β-catenin signaling pathway in human cancer cells [25]. Finally, gamma secretase inhibitors and sorafenib may also target activated CTNNB1 or Wnt pathways [26–28]. Gamma-secretase cleaves intracellular Notch, resulting in Notch activation; gamma secretase inhibitors can be effective at controlling disease growth in desmoid tumors, the vast majority of which have *CTNNB1* mutations [26]. Sorafenib may also decrease CTNNB1 class-specific, Wnt-pathway activation [27, 28].

#### AXIN1

Approximately 5–19% of liver cancer specimens harbor *AXIN1* mutations [20–22]. *AXINI,* by controlling the level of β-catenin, serves as a negative regulator of Wnt/β-catenin signaling [29]. Overexpression of wild-type *AXIN1*, but not mutant *AXIN1,* suppressed the proliferation of HCC cell lines and accelerated their programmed cell death, implicating AXIN1 as a therapeutic target in HCC [29]. Supporting this concept is the observation that expression of the wild-type *AXIN1* gene by adenovirus-mediated gene transfer promoted apoptosis in HCC cells, which had accumulated β-catenin as result of *APC*, *AXIN1,* or *CTNNB1* gene mutations [30]. AXIN stabilization by suppressing the poly-ADP-ribosylating enzymes tankyrase 1 and tankyrase 2 with the small molecule inhibitor XAV939 has been suggested as a novel way to target the Wnt pathway [31]. Finally, non-steroidal anti-inflammatory agents may inhibit the Wnt/ β-catenin signaling pathway in human cancer cells [25].

### P53 pathway

#### TP53

Approximately 13–48% of liver cancers harbor *TP53* mutations [20–22]. TP53 acts as a tumor suppressor in tumors; it induces growth arrest or apoptosis depending on the physiological settings and cell type [23]. Genomic aberrations in the p53 pathway are the most frequent abnormalities in diverse cancers and often correlate with high-grade histology [22]. *TP53* mutations in HCC in afflicted patients in Western countries are also linked with worse clinical stage and prognosis [21]. The *TP53* gene regulates multiple biologic processes, including the expression of VEGF-A [32]. Of importance in this regard, anti-angiogenic agents, such as bevacizumab, were associated with improved progression-free survival (PFS) in patients whose tumors harbored *TP53* mutations [33, 34]. In addition, it has been postulated that malignances with *TP53* mutations may be sensitive to Wee1 inhibitors, which are in clinical trials [35, 36].

### Chromatin remodeling

#### ARID1A

Approximately 4–17% of HCCs harbor *ARID1A* alterations [20–22, 37]. *ARID1A* participates in transcriptional activation and repression of select genes by chromatin remodeling [23]. *ARID1A* alterations were significantly more common in HCC related to alcohol abuse than in tumors of other etiology and demonstrated a correlation with *CTNNB1* mutations [20]. Samartzis and co-investigators reported that PI3K/AKT-pathway activation is also a crucial mechanism in *ARID1A*-mutated cancers and, consequently, *ARID1A*-deficient tumors show preclinical sensitivity to treatment with PI3K/Akt/mTor pathway inhibitors [38].

#### ARID2

Approximately 5% to 7% of liver cancers harbor *ARID2* mutations [18, 21, 37]. ARID2 is implicated in transcriptional activation and suppression of select genes by chromatin remodeling [23]. *ARID2* is an important tumor suppressor gene, but targeting it is challenging [39]. *ARID1A* and *ARID2* are components of the SWItch/Sucrose Non-Fermentable (SWI/SNF)-related chromatin remodeling complex. Better comprehension of the network of molecules involved in the SWI/SNF complex is required [40].

### Cell cycle

#### CDKN2A

Approximately 8% of HCCs harbor *CDKN2A* deletions [18, 20]. *CDKN2A* is a tumor suppressor gene that induces cell cycle arrest in G1 and G2 phases; it also suppresses the oncogenic action of CDK4/6 and MDM2 [23]. In the presence of *CDKN2A* mutations, CDK4 and CDK6 expression are up-regulated. *CDKN2A* inactivation has been correlated with poor prognosis independently of other traditional factors; in addition, *CDKN2A* alterations are discerned in more advanced, aggressive cancer [18]. Given the frequent loss of *CDKN2A* in HCC, CDK4/6 inhibitors are being tested in advanced HCC [13].

#### CCND1, FGF3, FGF4 or FGF19

Approximately 5% to 7% of liver cancer specimens harbor alterations of CCND1; 4% to 6% have alterations in *FGF3*, *FGF4*, or *FGF19* genes [18, 41]. Amplification of *FGF3/ FGF4/FGF19/CCND1* (all of which reside on the same amplicon on the long arm of chromosome 11) is associated with poor prognosis in resected HCCs, independent of classical prognostic variables [18]. Clonal growth and tumorigenicity of HCC cells harboring the 11q13.3 amplicon were selectively suppressed by an anti-FGF19 antibody as well as by RNAi-mediated knockdown of *FGF19* or *CCND1 *[42]. It is plausible that 11q13.3 amplification is a biomarker for patients most likely to respond to anti-FGF/FGFR agents [42].

Lenvatinib, an oral tyrosine kinase inhibitor targeting FGFR1–4, VEGFR1–3, KIT RET, and PDGFR-beta, has induced partial responses in a small subgroup of patients with HCC [43]. Not unexpectedly, when given to unselected patients with HCC, the FGFR inhibitor brivanib did not improve survival [44]. Dovitinib (another FGFR inhibitor) was also not superior to sorafenib in unselected patients with advanced HCC [45]. These trials suggest that studies with patient selection for the presence of the target gene are needed.

### Other genomic alterations:

#### TERT

*TERT* promoter mutations were recently identified as the most frequent somatic genomic defect in HCC, with an overall frequency of 60% to 90% [46–48]. Telomerase is a ribonucleoprotein enzyme that is critical for the replication of chromosome termini in most eukaryotes [23]. Telomerase overexpression is a key component of the transformation process in many cancer cells [23]. TERT extends telomeres and *TERT* mutations activate this activity, allowing cells to continue dividing. *TERT* alterations are one of the earliest genomic anomalies involved in malignant transformation in HCC and may, therefore, be considered as a tumor “gatekeeper.” [46] Tahtouh and colleagues demonstrated that telomerase inhibition decreases alpha fetoprotein (AFP) expression in individuals with HCC and may have a suppressive effect on cell growth via attenuation of telomere repair [49]. Unfortunately, low-dose cyclophosphamide administration followed by GV1001 vaccinations (GV1001 being a telomerase vaccine) did not demonstrate efficacy [50]. Several other approaches are being studied in the development of vaccines that may induce TERT immunogenicity [51]. Other telomerase-targeting compounds, such as small molecule inhibitors RHPS4 and BRACO-19, and also PARP inhibitors, which lead to a rapid decrease in median telomere length, are innovative strategies [51–53].

#### RPS6KA3

About 4% to 7% of liver cancer tissues bear *RPS6KA3* mutations [18, 22]. In part by modulating mTOR signaling, RPS6KA3 mediates stress-induced and mitogenic activation of transcription factors and cellular differentiation, growth, and survival [23]. In addition, RPS6KA3 (RSK2) is a kinase enzyme that acts downstream of the MAPK/ERK pathway [40]. RPS6KA3 tends to be altered in poorly differentiated HCC [54]. Taking into account its dual function (regulation of the MAPK/ERK and mTOR signaling), RPS6KA3 mutations may be difficult to target [40].

Other functions for RPS6KA3 may also be operative. For instance, by building a genome-wide miR-191 target set, Polioudakis et al. were able to identify and validate RPS6KA3 as a direct target of miR-191 [55]. MicroRNAs are a class of small non-coding RNAs that act as post-transcriptional gene regulators. miR-191 is abnormally expressed in over 20 types of cancer and in protean other diseases, e.g., Crohn’s, diabetes-type 2, Alzheimer’s, and pulmonary hypertension. By modulating important transcription factors, miR-191 regulates a variety of cellular processes: chromatin remodeling, cell cycle, differentiation, proliferation, migration, and apoptosis.

Other activities may also be relevant to perturbed RPS6KA3. For instance, knockdown of histone deacetylase 2 (HDAC2) induces RPS6KA3 repression and offers preclinical proof-of-concept for HDAC2 blockade in HCC [56].

#### PTEN loss or alterations

PTEN loss by immunohistochemistry can be seen in up to 53% of patients diagnosed with HCC [57]. *PTEN* mutation or deletion (both of which are mechanisms that lead to loss of PTEN expression) may be discerned in a smaller proportion of patients (~20 to 30%) [58, 59]. Loss of PTEN activates the PI3k/Akt/mTor axis and may be druggable by PIK3CA, AKT or mTor inhibitors. A phase 3 trial of the mTor inhibitor everolimus demonstrated no survival benefit for patients with advanced HCC who had failed sorafenib; however, the participants were not selected by PTEN or other PI3K-related abnormalities. Further investigation with appropriate biomarker selection is warranted [60].

### Epigenetics, MicroRNA (miRNA), long non-coding RNA and HCC

Aberrant epigenetic silencing of tumor suppressor genes by promoter DNA hyper-methylation and histone deacetylation plays an important role in carcinogenesis. The potential reversibility of these epigenetic abnormalities makes targeting them with drugs that modify chromatin an attractive therapeutic approach [61].

In hepatic carcinogenesis, studies have shown that alterations in epigenetics and miRNA correlate with the progression from precancerous lesions to HCC [61, 62]. miRNA are small (~22 nucleotide) non-coding RNAs that regulate expression of diverse target genes [63]. In HCC, tumor suppressor genes may be epigenetically silenced by histone modifications, such as methylation of histone H3 lysine 9 (H3K9) and tri-methylation of H3K27, as well as DNA hyper-methylation of CpG island promoters and histone deacetylation [61, 62, 64, 65]. In addition, oncogenes that may normally be inactive because of specific suppressor miRNA may be activated if the miRNA is epigenetically silenced [63]. Restoring the expression of tumor suppressor miRNA or tumor suppressor genes by inhibitors of DNA methylation and histone deacetylase may therefore be a rational treatment approach for HCC.

Recently, long, non-coding RNAs have also been found to be differentially expressed in HCC and have been implicated in HCC pathogenesis [66]. Non-coding RNAs were once thought to be transcriptional “noise”. However, accumulating investigations have shown these molecules to play significant regulatory roles. Long non-coding RNAs (over 200 nucleotides in length) are endogenous cellular RNA molecules that are incapable of being translated into proteins. They have a significant role in epigenetic regulation since they modulate gene expression by mechanisms including chromatin modification and genomic imprinting. In HCC, long, non-coding RNA have been implicated in initiation, progression, and metastasis. However, research on these molecules in this disease remains in its infancy, as does how to exploit them for therapeutic purposes.

### Angiogenesis and HCC

HCC is a hypervascular malignancy mostly supplied by hepatic arteries; in contrast, in normal liver parenchyma, regenerative and dysplastic nodules are generally supplied by the portal vein [67]. On microscopic exam, HCC shows marked vascular anomalies, arteriogenesis and capillarization. Vascular endothelial growth factor (VEGF) and angiopoietins are important endothelium-specific growth factor families in HCC. Sorafenib is a multikinase suppressor that inhibits tumor cell growth and angiogenesis by inhibiting VEGF receptor. Sorafenib has been approved for treatment of HCC based on a randomized controlled trial that showed a ~ 2.5 month survival advantage, albeit with response rates less than 5 %.

### Immunotherapy in HCC

Immunotherapeutics are very promising therapeutic tools in many advanced cancers, especially those that are virally induced. HCC is therefore an attractive candidate for immunotherapy. Recently, immune checkpoint inhibitors, such as ipilimumab (CTLA4 inhibitor) and nivolumab (PD-1 inhibitor), demonstrated survival benefits in multiple tumor types, including melanoma, non-small cell lung cancer, and renal cell carcinoma [64, 65, 68, 69]. Programmed cell death protein 1 (PD-1) and its ligands--programmed cell death 1 ligand 1 (PD-L1) and 2 (PD-L2)--play important roles in regulating immune responses PD-L1 was expressed in 82% of HCC specimens [70]. PD-L1 expression was higher in hepatitis B-positive patients than in those that were negative for this virus. A phase I/II study of nivolumab in patients with advanced HCC demonstrated a 19% response rate (including 5% complete responses) [71, 72]. Importantly, some responses were durable. Of note, in many malignancies, the presence of PD-L1 is associated with response to anti-PD-1/PD-L1 agents (0–17% response rate in PD-L1-negative versus 36–100% response rate in PD-L1-positive patients) [73]. From a mechanistic perspective, it is of interest that PD-L1 is induced in hepatocytes by viral infection [74].

Other immunotherapeutic approaches are also worth mentioning. Oncolytic viruses offer the promise of selective cancer treatment via direct and immune-mediated mechanisms. The premise of oncolytic viruses lies in their preferential infection of cancerous cells [75]. In HCC, several oncolytic viruses have been studied in Phase I and II protocols [75]. Results from a phase 1 clinical trial of intratumoral JX-594 in patients with refractory liver tumors demonstrated mechanistic proof-of-concept, safety, and antitumor efficacy for JX-594 replication and systemic dissemination to distant metastases [76]. Of note, three HCC patients had tumor marker response (between 56% and 98% reduction), and one participant had a confirmed radiographic partial response. In a Phase II dose-finding clinical trial, JX-594 was administered at two doses (low-dose (10^8^ PFU) and high-dose (10^9^ PFU) of JX-594). The study was terminated early as patients receiving higher doses of JX-954 showed a significant longer overall median survival (14.1 months in the high-dose group versus 6.7 months in the low-dose group) [77]. Additional viruses that are in clinical trials include adenoviruses such as ONYX-015 as well as a vesicular stomatitis virus, which encodes the human IFN-β gene (VSV-hIFN-β) (NCT01628640) [78]. ONYX-015 was effective in killing cells and suppressing HCC cell proliferation both in vitro and in vivo. The cell killing was more prominent in cells possessing abnormal p53 [79].

Therapeutic regimens that counteract the immunosuppressive mechanisms have the potential to dramatically alter clinical outcomes of HCC. Currently, the multi-targeted kinase inhibitor sorafenib, which improves survival by 2.3–2.8 months, is the standard of care for patients with advanced HCC [15, 80, 81]. There is an urgent need for new, molecularly or immune-targeted agents that would improve clinical outcomes [81, 82]. Antibody-mediated blockade of PD-1 has demonstrated therapeutic benefit in the setting of refractory solid tumors, while inhibition of Tim-3 signaling in pre-clinical models helped to reestablish anti-tumor T cell activity [83, 84]. Immunotherapy affecting angiogenesis and/or the tumor vasculature portrays an effective modality based on the potential to generate long-lived anti-vascular T cell responses. Pre-clinical data from Niethammer and coworkers indicated that an anti-VEGFR2 vaccine re-establishes an autoimmune response against self antigens and blocks tumor dissemination through a T cell-dependent process [85]. VCAM-1 also plays an important role in trapping activated T cells in the liver microvasculature, which modulates HBV- and HCV-related hepatic carcinogenesis and may provide unique treatment targets for HBV- and HCV-associated HCCs [86]. Lizuka et al. found up-regulation of NK cell-associated killer cell immunoglobulin-like receptors (KIRs) in HCV-infected (and not HBV-infected) HCC patients [87]. Importantly immunotherapy needs to be tailored to individual patients based on the discrete etiologies for their HCCs [81].

## Conclusions

### Perspective and future directions

HCC is a difficult-to-treat and lethal malignancy. Chemotherapy did not prolong overall survival in HCC, and there is an unmet need to develop novel therapeutics for this illness. The emergence of genomic- and immune-based therapies is transforming treatment of many cancers and is beginning to be applied to HCC.

Of note, there are several genomic mutations that are common in HCC. The *TERT* promoter is mutated in approximately 60% to 90% of patients, and remains a challenge to target. Alterations in *TP53* are seen in up to 40% of HCC patients. CTNNB1 is a negative regulator of the Wnt pathway, and often altered in HCC. *FGF* anomalies, as well as alterations in the cell cycle machinery and PTEN loss, are frequently observed. Some of these abnormalities may be pharmacologically tractable by approved agents or by specific inhibitors that are under development.

Novel immune checkpoint inhibitors are gaining momentum in other cancers such as melanoma and non-small cell lung cancer. In HCC, a phase II study with nivolumab in patients with Child-Pugh A HCC revealed encouraging results: 19% overall response rate and potential for durable response. The number of patients is small, and additional trials are in progress. Novel immune therapy, such as anti-PD-L1 or other immune-targeted agents, has the potential to change the landscape of HCC treatment in the future.

Although systemic therapy in HCC has been disappointing in the past, we are at the dawn of new era. More studies that utilize genomic profiling and biomarker-matched molecularly targeted therapy as well as immunotherapy, and combinations of the above, are needed in order to improve the prognosis of patients suffering from HCC and advance the field.
